# Supplementary figures and images for: Serial infection with SARS-CoV-2 Omicron BA.1 and BA.2 following three-dose COVID-19 vaccination
Source: Front Immunol. 2022 Sep 6;13:947021. doi: 10.3389/fimmu.2022.947021 (PMC9485663; doi:10.3389/fimmu.2022.947021)

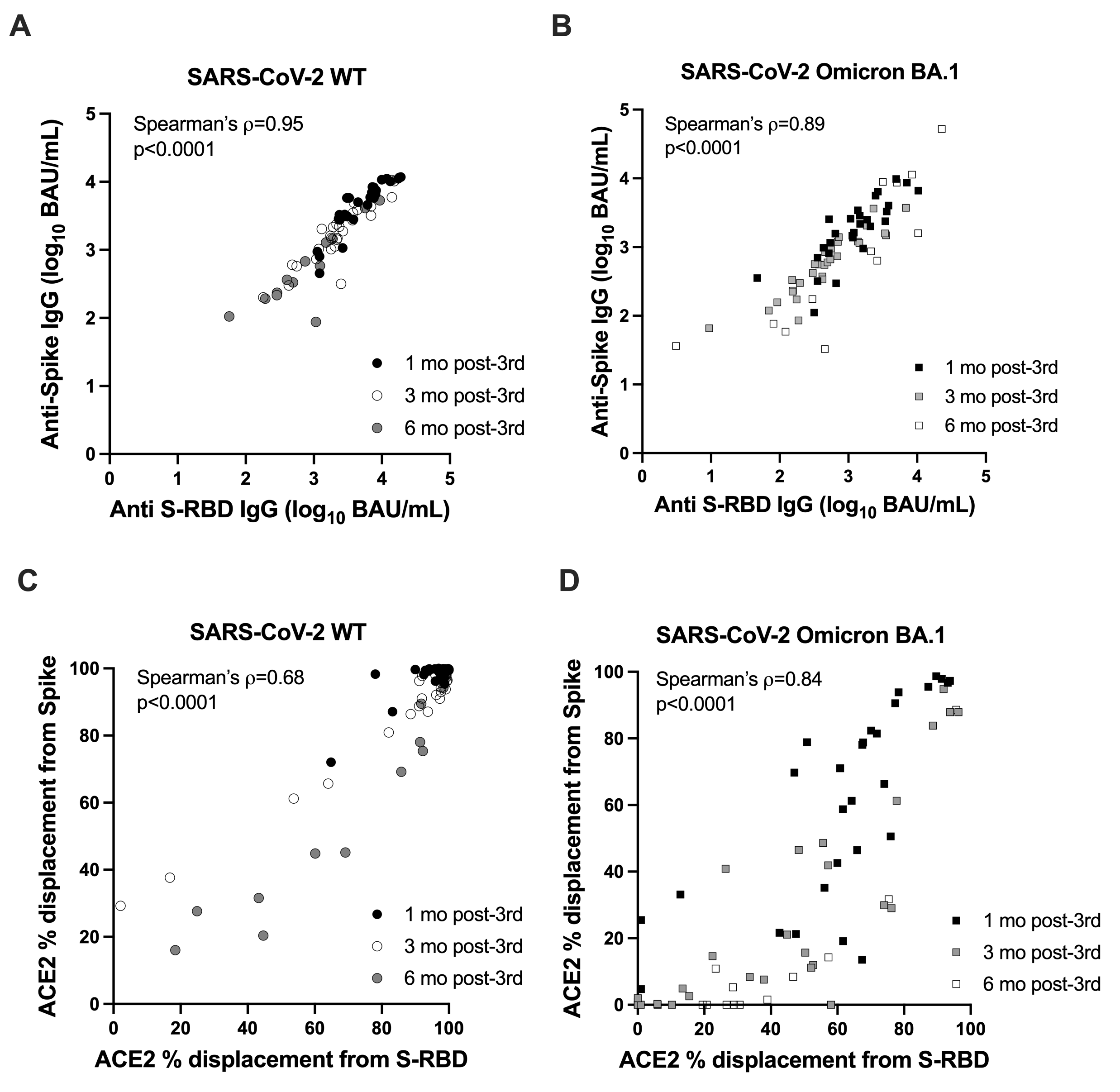

Supplement: Supplementary Figure 1 — Correlations between wild-type (WT) and BA.1-specific anti-S-RBD and anti-Spike humoral responses measured following three COVID-19 vaccine doses using Meso Scale Diagnostics V-plex panels 22 and 25. All participants who were evaluated for BA.2 responses (i.e. those shown in Figure 2 ) are included in this analysis. Symbols are colored based on post-vaccination time point, though the Spearman’s rho (ρ) and p-value reported are for the combined data. [file Image_1.tiff]
